# Pharmacokinetic Covariates Influencing Mycophenolate Area Under the Curve in a Danish Renal Transplant Population

**DOI:** 10.3390/pharmaceutics18050624

**Published:** 2026-05-20

**Authors:** Svend Buus, Eva Greibe, Lara Aygen Øzbay, Elke Hoffmann-Lücke, Niels Henrik Buus

**Affiliations:** 1 Department of Clinical Biochemistry, Aarhus University Hospital, 8200 Aarhus, Denmark; 2 Department of Clinical Medicine, Aarhus University, 8200 Aarhus, Denmark; 3 Department of Renal Medicine, Aarhus University Hospital, 8200 Aarhus, Denmark

**Keywords:** area under the curve (AUC), mycophenolate, mycophenolic acid, therapeutic drug monitoring (TDM), limited sampling strategy (LSS), renal transplantation, pharmacokinetics

## Abstract

**Background/Objectives:** Mycophenolic acid (MPA) monitoring may improve organ transplant outcomes, yet clinical implementation is hindered by the complex pharmacokinetics of MPA and a lack of clarity regarding the influence of specific patient factors on drug exposure. While the area under the curve (AUC) is the gold standard for MPA monitoring, it is not easily validated or implemented in routine practice. This pilot project aimed to identify key clinical and biochemical covariates driving pharmacokinetic variability in a renal transplant population. **Methods:** This prospective study analyzed 103 samples from 66 kidney transplant recipients. To estimate total drug exposure (AUC), a limited sampling strategy was used with plasma samples collected at trough, and then 30 and 120 min post-dose. We performed linear univariate and multivariate regressions to evaluate the impact of patient characteristics (age, sex, body mass index (BMI)) and biochemical measurements (P-albumin, P-creatinine, estimated glomerular filtration rate (eGFR), B-tacrolimus) on MPA-AUC, peak concentrations (C_max_) and trough levels. **Results:** At 750 mg twice daily, the median MPA-AUC was 43.5 mg·h/L (IQR: 34.5–53.5). After adjusting for dose, P-albumin and age were independent predictors of AUC: P-albumin levels were positively associated with AUC (*β* = 1.849, *p* < 0.001), while age showed a modest negative association (*β* = −0.282). BMI was significantly and inversely associated with trough concentrations (*β* = −0.137, *p* = 0.011), indicating that higher BMI is linked to lower trough concentrations. Male sex was associated with significantly lower AUC and C_max_ compared to females. Notably, eGFR and B-tacrolimus levels did not significantly influence MPA exposure in this cohort. **Conclusions:** The covariates BMI, sex, age, and P-albumin significantly influence MPA-AUC. LSS-based AUC monitoring, using 30–60 mg·h/L as a target and with consideration of a few patient-specific factors, could be a pragmatic and feasible approach to improve MMF dosing strategies in kidney transplant recipients.

## 1. Introduction

In kidney transplant recipients, maintenance of immunosuppressive therapy is essential to prevent rejection [1,2,3]. Over the past 25 years, mycophenolate mofetil (MMF) has been a key component of standard immunosuppressive regimens, usually in combination with a calcineurin inhibitor such as tacrolimus [4,5,6]. Following oral administration, MMF is rapidly absorbed from the gastrointestinal tract and hydrolyzed to its active metabolite, mycophenolic acid (MPA) [1,7,8]. MPA acts as a potent, selective, and reversible non-competitive inhibitor of inosine monophosphate dehydrogenase (IMPDH), the enzyme that catalyzes the conversion of inosine monophosphate (IMP) to xanthosine monophosphate (XMP), which is a rate-limiting step in de novo guanosine nucleotide synthesis [9,10]. Of all cells, T- and B-lymphocytes are most dependent on this pathway [9,11,12]. IMDPH exists in two isoforms, type I and type II [13], with type II being expressed mostly in activated lymphocytes. MPA is shown to inhibit the type II enzyme five times more than type I [9].

The pharmacokinetic profile of MPA demonstrates marked inter- and intra-individual variability [14]. MPA is extensively (>97%) bound to plasma proteins [7,15] and is primarily metabolized in the liver via uridine 5′-diphospho-glucuronosyltransferase (UGT), forming the inactive metabolite mycophenolic acid glucuronide (MPAG) [16,17]. MPAG is excreted predominantly by the kidneys via glomerular filtration [18] and may undergo enterohepatic recirculation, often resulting in a secondary plasma MPA concentration peak approximately 6–12 h post-dose [5,14,19,20]. Several studies have sought to elucidate this interindividual variability by examining the effects of different covariates on the body’s handling and metabolism of MPA and MPAG, with heterogeneous results. Multiple studies have demonstrated an association between MPA concentrations and the coadministration of other immunosuppressive agents, such as ciclosporin and tacrolimus [21,22,23]. Other studies support that organ functions, including renal and hepatic function, influence MPA clearance and, consequently, overall exposure [21,24,25]. In addition, it has been shown that individual characteristics, such as age, body weight, and sex, also affect drug exposure [18,26,27].

While MMF was initially introduced as a fixed-dose treatment [1,28,29,30], subsequent practice has shifted toward dose adjustments based on time post-transplant, clinical effects, and management of adverse events. However, the substantial pharmacokinetic variability of MPA makes optimal dosing strategies an ongoing unresolved issue [31,32,33]. Current consensus supports therapeutic drug monitoring (TDM) as the preferred approach for individualizing MMF therapy [1,4,33,34,35]. The ideal method for TDM of MPA is measurement of the area under the curve (AUC) from 0 to 12 h post-dose (AUC_0–12h_) [4,36]. This method ideally involves hourly serial sampling over the full 12 h dosing interval. However, such intensive sampling is impractical in routine clinical care due to costs, logistical challenges, and patient inconvenience. Consequently, in aiming to find a method that both considers feasibility and accuracy, Filler & Mai [37] were the first to propose a method for calculating an AUC using limited samplings. This resulted in broad research focusing on developing limited-sampling strategies (LSS) to estimate AUC_0–12h_ [38,39,40,41,42]. One LSS model that proved to be more concise when trying to estimate AUC_0–12h_ has been validated using three plasma concentrations obtained at pre-dose, 30 min, and 2 h post-dose (AUC_0–2h_) [5].

Optimizing immunosuppressive management requires moving beyond standardized dosing toward individualized therapy. This study investigates the pharmacokinetic profile of MPA in a Danish kidney transplant population to identify key covariates influencing MPA exposure using an established model to determine MPA-AUC. We further assess the feasibility of performing LSS blood sampling in routine clinical practice.

## 2. Methods

### 2.1. Study Design

The study was designed to be non-interventional and observational, with prospective blood sample collection and retrospective data analysis as part of a quality assessment aimed at evaluating the relevance of various covariates on MPA-AUC. The dataset was used for subsequent implementation of MPA-AUC calculations into routine clinical practice. MPA concentrations were blinded to clinicians and patients throughout the study period and were not used for clinical management.

### 2.2. Patients

In total, 74 patients who underwent kidney transplantation at the Department of Renal Medicine at Aarhus University Hospital from 1 September 2024 to 30 June 2025 were enrolled. The study involved no intervention or deviation from standard clinical care and used only data collected as part of routine practice. Patient data were handled confidentially in compliance with data protection legislation. Data collection adhered to local and national regulations and was approved by the hospital board.

### 2.3. MMF Treatment Strategy

Patients received twice-daily doses of MMF, with an initial dose typically set at 750 mg; all patients were treated with mycophenolate mofetil (Myfenax^®^ Teva capsules of 500 mg or 250 mg, Teva Denmark A/S, Søborg, Denmark) and none received mycophenolate sodium. Concomitant immunosuppressive therapy included tacrolimus and prednisolone. The patients were not fasting on the day of MPA-AUC determination, and they were instructed to take their other medications as usual. The calculated MPA-AUC values were concealed from the clinicians, who adjusted treatment according to standard clinical fixed-dose protocols. If the post-transplant period was without complication, the usual practice was to reduce the dose to 500 mg bidaily after six weeks.

### 2.4. Biochemical Analytical Method

To evaluate the feasibility and utility of estimating the AUC_0–2h_ for MPA in renal transplant recipients by TDM, three sequential blood samples were analyzed at two post-transplant time points: at 2–3 weeks and at 8–10 weeks post-transplant. On the day of sampling, a baseline sample was collected prior to MMF administration. The exact time of dosing was recorded, and additional samples were obtained 30 (C_30_) and 120 (C_120_) minutes post-dose.

Blood samples were collected in EDTA tubes, then centrifuged and pipetted within 3 h. The samples were then kept frozen at −20 °C until analysis, which occurred every 14 days.

The concentration of MPA in plasma was determined using a commercially available LC-MS/MS kit (MassTox^®^ TDM Series A Parameter Set Mycophenolic Acid in Serum/Plasma, Chromsystems, Munich, Germany). Sample preparation and chromatographic and mass spectrometric analysis were performed following the manufacturer’s instructions. In brief, plasma extracts were analyzed by liquid chromatography coupled with tandem mass spectrometry (LC-MS/MS) using multiple reaction monitoring (MRM) in positive ionization mode. The analysis was performed at the accredited hospital laboratory (ISO 15189 [43]) of Department of Clinical Biochemistry at AUH, Denmark. The analysis was run on an Agilent 1290 HPLC Infinity system coupled to an Agilent 6490 Triple Quad mass spectrometer (Agilent Technologies, Santa Clara, CA, USA). Precision of the analysis was based on two controls, one at a low concentration (C_1_: 1.7 ± 0.27 mg/L) and one at a higher concentration (C_2_: 5.36 ± 0.80 mg/L), which, during the timespan of testing, were 6.5% and 5.6%, respectively.

Other relevant biochemical parameters were all analyzed as follows: P-creatinine and P-albumin on Atellica CH (Siemens Healthcare Diagnostics, Ballerup, Denmark); and B-tacrolimus on Agilent HPLC Infinity System coupled to an Agilent 6495 Triple Quad mass spectrometer (Agilent Technologies, Santa Clara, CA, USA). eGFR was subsequently calculated (Male: 141 · (P-creatinine/(0.7 · 88.4))^−0.329^ · 0.993^age^, Female: 144 · (P-creatinine/(0.7 · 88.4))^−0.329^ · 0.993^age^).

### 2.5. Data Calculations and Statistical Analysis

The blood test results were collected within the laboratory information system (LABKA II), which processed and organized the data. The three plasma concentrations were calculated using the LSS model previously validated by Pawinski and Kuypers [4,5]: AUC_0–2h_ = 7.75 + (6.49 · C_00_) + (0.76 · C_30_) + (2.43 · C_120_). Once processed, the data was extracted as a structured dataset for further analysis. The choice of formula is based on a previous Scandinavian multicenter study [27] and further discussed in Section 4.

Statistical analyses were performed using RStudio (R version 2025.05.0 Build 496; R Foundation for Statistical Computing, Vienna, Austria). Associations between the outcome and possible explanatory covariates were assessed using linear regression models fitted with the lm() function in R. Univariable analyses were conducted first, followed by multivariable linear regression models adjusted for dose. Regression coefficients are reported as β estimates with corresponding 95% confidence intervals (CIs). Explained variability were assessed using the coefficient of determination (R^2^), representing the proportion of variance in MPA explained by the singular covariates. To maintain the integrity of the analyses, only validated and complete data were included in the statistical evaluations.

## 3. Results

### 3.1. Patient Characteristics and Exclusions

**Figure 1:**
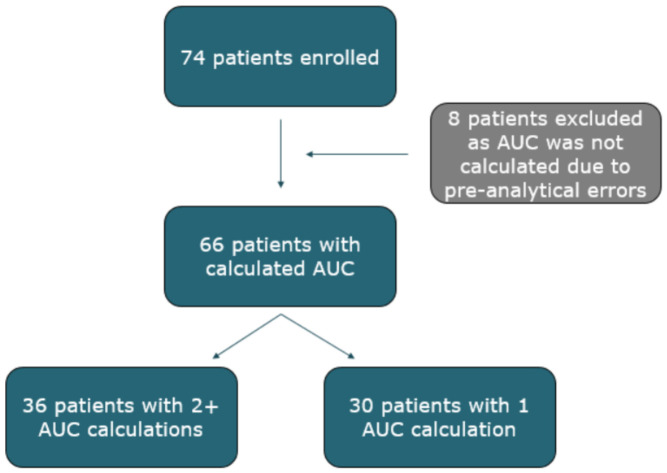
Flowchart of the included patients.

A flowchart depicting patient inclusion is presented in Figure 1. In total, data from 74 patients were used in the study. Eight patients were excluded due to pre-analytical errors that precluded AUC calculation. Among the remaining 66 patients, AUCs were obtained only once for 30 individuals during the period of data collection.

Baseline demographic and clinical characteristics of the cohort are presented in Table 1. In addition to MPA, blood samples were analyzed regarding the parameters as described in Table 1 on the day of sampling. The time of blood sampling was carefully documented and strictly controlled. Samples taken outside a predefined acceptable time window were excluded from the analysis to ensure consistency and validity of the pharmacokinetic assessments. All covariates, including dose, were recorded at the time of sampling. Samples from the same patient under different dosing regimens were treated as separate time-specific observations with their respective covariate profiles. Clinical variables were obtained through review of the electronic patient journal.

### 3.2. Total Sample Size

A total of 103 sample sequences were collected from 66 patients, and MPA-AUC was successfully calculated for each sample sequence. Of the 103 samples, 38 were associated with a twice-daily 500 mg dose, 60 with a twice-daily 750 mg dose, and five with a twice-daily 1000 mg dose.

### 3.3. Concentrations Based on Time and Dose

**Figure 2:**
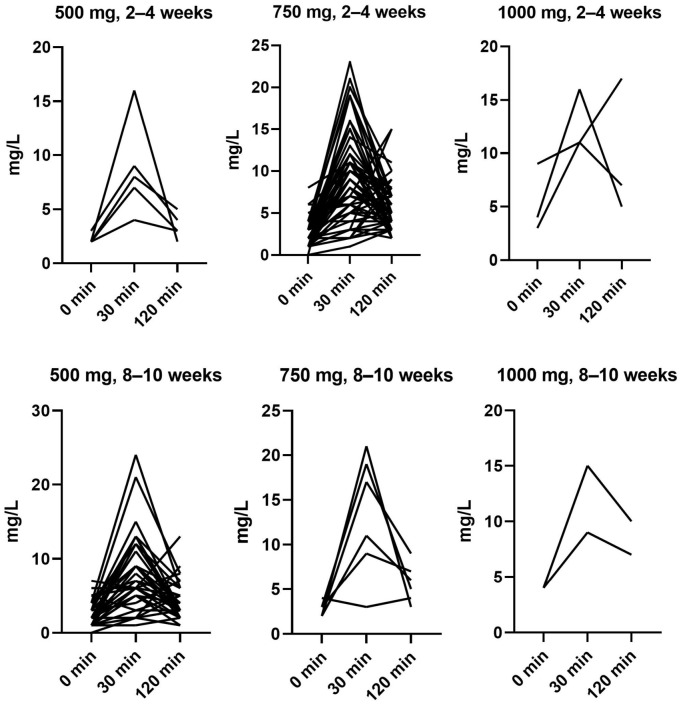
Individual measurements of P-mycophenolate (mg/L) stratified by dose and time of sampling.

Figure 2 illustrate the plasma concentration profiles of MPA for each individual blood sample. Among the 103 AUC calculations, the largest subgroup (N = 54) comprised early post-transplant samples (2–4 weeks) from patients receiving 750 mg twice daily. Another major subgroup consisted of patients receiving 500 mg twice daily at 8–10 weeks post-transplant (N = 33). The expected concentration–time profile is characterized by an initial ascending phase, reflecting systemic absorption and distribution, followed by a descending phase driven by elimination processes. Specifically, concentrations are anticipated to increase dynamically from trough to a peak level at C_30_, corresponding to the time of maximal systemic exposure (C_max_). Thereafter, concentrations are expected to decline progressively toward near-trough levels at C_120_. Contrary to expectations, 25 (24%) of the analyses demonstrated a C_max_ at C_120_ rather than at C_30_. No factors could be identified to explain the occurrence of late peak values, and the pattern was not reproducible in patients who underwent multiple AUC assessments.

### 3.4. AUC Levels Based on Dose

**Figure 3:**
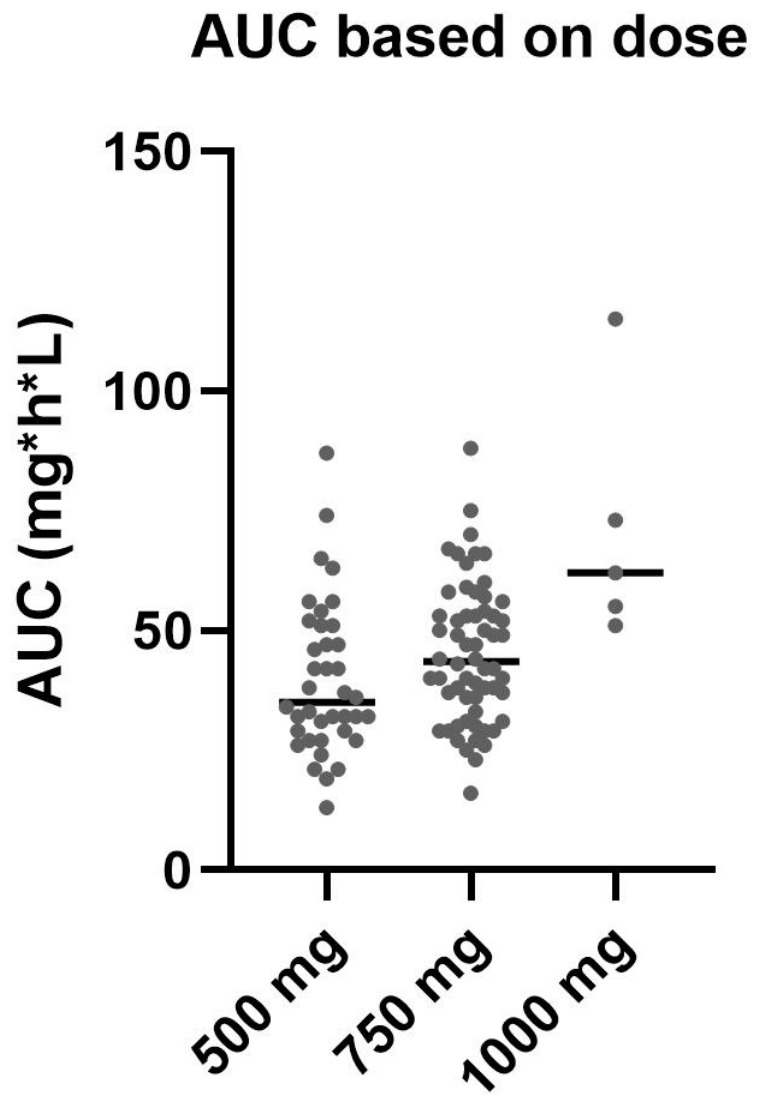
Boxplots of AUC calculations (mg·h/L) based on dose: N = 38 samples with 500 mg (median: 35 mg·h/L, IQR: 29–51), N = 60 with 750 mg (median: 43.5 mg·h/L, IQR: 34.5–53.5), and N = 5 with 1000 mg (median: 62 mg·h/L, IQR: 53–94).

Figure 3 presents boxplots of AUC levels stratified by the three commonly administered twice-daily MMF doses. The median MPA-AUC was lowest in 500 mg (AUC: 35 mg·h/L, IQR: 29–51). At 750 mg, the median MPA-AUC was 43.5 mg·h/L (IQR: 34.5–53.5). In the 1000 mg group, the median MPA-AUC was 62 mg·h/L (IQR: 53–94). The interquartile range was widest in the 1000 mg group, reflecting greater variability, likely attributable to the small sample size.

### 3.5. Influences of Covariates on MPA-AUC

Univariate regressions on individual MPA samples (C_00_, C_30_, and C_120_) were performed, as well as dose-adjusted multivariate regressions on the other covariates, to investigate their influences. Results are shown in Table 2.

In dose-adjusted analyses, C_0_ (trough concentration) and C_120_ levels were strongly associated with MPA-AUC, explaining 60–83% of the variability. C_30_ also contributed significantly, though the effect was smaller (R^2^~0.23). Among other covariates, P-albumin and age demonstrated independent effects: higher P-albumin was associated with increased AUC, whereas older age showed a modest negative association. BMI and body weight showed trends toward lower AUC with increasing values, but only BMI approached statistical significance. Male sex was associated with a significantly lower total MPA-AUC compared with females. Renal function (P-creatinine and eGFR), B-tacrolimus, and weeks post-transplant did not significantly influence MPA-AUC after adjusting for dose. Overall, dose was the strongest determinant of MPA-AUC, while patient characteristics contributed modestly.

### 3.6. Influences of Covariates on C_0_

Dose-adjusted multivariate regression analyses were performed to assess the influence of patient covariates on trough concentration of P-MPA. Results are shown in Table 3.

After adjusting for dose, P-albumin and BMI were significant predictors of P-MPA, whereas eGFR, B-tacrolimus, weight, P-creatinine, sex, and age showed no statistically significant association.

P-albumin showed a positive association with trough concentration (β = 0.177, 95% CI: 0.095 to 0.258 mg/L, *p* < 0.001), indicating that higher albumin levels are linked to higher trough concentrations. BMI was inversely associated (β = −0.137, 95% CI: −0.241 to −0.033 mg/L, *p* = 0.011), suggesting that higher BMI is associated with slightly lower trough concentrations. Dose remained a significant positive predictor across all models (Beta ~0.002, *p* = 0.040), confirming pharmacokinetic behavior as expected.

Overall, the included covariates explained a modest proportion of the variability in trough concentrations (adjusted R^2^~3–18%), indicating that additional factors beyond the measured clinical covariates may contribute to inter-individual variability.

### 3.7. Influences of Covariates on C_30_

Results of multivariate regression of covariates for the 30 min P-MPA are shown in Table 4.

In analyses of dose-adjusted C_30_ values, most covariates—including eGFR, B-tacrolimus levels, weight, BMI, P-albumin, and age—did not show statistically significant effects. P-creatinine, sex, and donor organ status were the covariates with significant associations. Higher P-creatinine levels were linked to slightly lower C_30_ (β = −0.019, 95% CI −0.036 to −0.003 mg/L, *p* = 0.021). Male sex was associated with a significantly lower C_30_ compared with females (β = −3.480, 95% CI −5.660 to −1.300 mg/L, *p* = 0.002). Deceased donor transplantation was associated with a significantly lower C_30_ compared with living donors (β = −2.500, 95% CI −4.580 to −0.420 mg/L, *p* = 0.019).

The single-variable dose model suggested a borderline positive effect of MMF dose on C_30_ (β = 0.008, 95% CI −0.000 to 0.015 mg/L, *p* = 0.052). Overall, these results indicate that, after adjusting for dose, demographic and laboratory parameters have limited predictive value for C_30_ of MPA.

## 4. Discussion

This study investigated the influence of clinical and biochemical covariates on MPA pharmacokinetics in a renal transplant population, and we found significant effects of sex, BMI, age, and P-albumin on MPA-AUC.

Our findings showed that males present significantly lower MPA-AUC than females. This observation is consistent with findings from previous pharmacokinetic investigations. In a cohort of 67 stable renal transplant recipients, Johnson et al. [44] reported significantly higher MPA-AUC_0–12h_ in female patients compared with males. Moreover, after normalization for BMI, females demonstrated slower MPA clearance than males. Similarly, a study conducted by Staatz et al. [45] among 147 stable renal transplant recipients, females exhibited significantly higher MPA-AUC values. In that analysis, the sex difference in exposure was further supported by higher trough MPA concentrations in females; however, but this specific finding was not reproducible in our dataset.

The observed sex-related differences in MPA exposure may be explained by shared metabolic pathways between MPA and estrogens, as well as their similar binding affinity for uridine glucuronosyltransferase 1A (UGT1A), the enzyme responsible for MPA glucuronidation. Competition or modulation at the level of UGT1A could contribute to reduced clearance and consequently higher systemic exposure in female patients [46]. Together, these findings reinforce the potential clinical relevance of sex as a determinant of MPA pharmacokinetics in renal transplant recipients.

Our findings demonstrated an inverse correlation between MPA-AUC levels and BMI, indicating that patients with higher BMI had lower systemic exposure to MPA. The influence of body weight and BMI on MPA pharmacokinetics has been examined in previous investigations with comparable results. In a cohort of 43 Japanese renal transplant recipients, Yamada et al. [47] reported that dose-adjusted MPA-AUC decreased with increasing body weight, suggesting that fixed dosing may result in lower exposure. Similarly, Nourbakhsh et al. [27], in a study including 210 renal transplant recipients, reproduced the inverse association between body size and MPA exposure. In our cohort, body weight alone showed only a non-significant trend toward an inverse association with MPA-AUC. However, when BMI was examined instead of absolute body weight, the relationship reached statistical significance. This finding may suggest that BMI, as a composite measure incorporating both weight and height, more accurately reflects distributional or metabolic factors influencing MPA pharmacokinetics than body weight alone.

The influence of age on MPA-AUC has previously been explored in renal transplant populations. Tang et al. [48] compared elderly patients (≥60 years) with younger (<59 years) patients, and only modest, non-significant differences in MPA exposure were observed between the two groups. Similarly, in the pharmacokinetic profiling study by Shum et al. [49], age was evaluated as a potential covariate but was not retained as a major determinant of apparent MPA clearance in the final pharmacokinetic model. In contrast to these findings, our results demonstrated a significant inverse association between age and MPA-AUC, indicating lower systemic exposure with increasing age. This observation is not consistently supported by previous pharmacokinetic research and therefore warrants cautious interpretation. Differences in study design, sampling strategy, population characteristics, or adjustment for confounding factors such as albumin and renal function may partly explain the discrepancy.

Interestingly, while pharmacokinetic studies generally report only modest age-related differences in MPA exposure, clinical outcome studies suggest that elderly transplant recipients experience a lower risk of acute rejection. One study [50] reported reduced rates of acute rejection in older recipients, supporting the concept of immunosenescence and the potential need for age-adapted immunosuppressive strategies. Together, these findings highlight the complex interplay between age, pharmacokinetics, and immune responsiveness, suggesting that chronological age alone may not fully capture the biological factors influencing MPA exposure and clinical outcomes.

MPA is highly protein bound (>97%), making plasma albumin a key determinant in its pharmacokinetics. Hypoalbuminemia increases the unbound fraction, facilitating faster clearance. Our data supported a significant positive influence of P-albumin on MPA-AUC. In our cohort, 9.6% of AUC measurements were performed in patients with P-albumin < 32 g/L, with the lowest measured value at 28 g/L. These findings underscore the importance of monitoring P-albumin when interpreting MPA exposure or adjusting doses, even for small dose reductions within the lower reference range.

Therapeutic drug monitoring using AUC-based strategies such as LSS is increasingly adopted in transplant centers, and it has been linked to improved outcomes in renal transplant recipients [35,51]. However, no universally recommended AUC target exists across organ types, reflecting variability in pharmacokinetics, formulations, concomitant calcineurin inhibition (CNI) therapy, and patient populations [52]. An MPA-AUC range of 30–60 mg·h/L is commonly applied in kidney transplant recipients early after transplantation, especially in combination with tacrolimus. While therapeutic intervals are frequently employed to assess clinical outcomes, this was beyond the intended scope of the present study. Further research is required to evaluate the clinical impact of dose-adjusted regimens guided by TDM.

Therapeutic drug monitoring of MPA in clinical practice requires a limited sampling strategy (LSS), with three- or four-sample protocols over 2–4 h being most practical. A three-sample, 2 h LSS, as proposed by Pawinski [5], appears to reflect the full 12 h AUC more accurately than other approaches and has been adopted in several Scandinavian laboratories [27], even though the original formula was based on a relatively small patient cohort. To enhance clinical feasibility in our study, we focused on a 2 h, three-sample LSS. Although cross-validation against other formulas was not performed, a recent Danish study [53] found several three-sample, 2 h LSS formulas fit well with the full 12 h AUC measurements (R^2^ 0.85–0.90), supporting the approach. Overall, a 2 h, three-sample LSS appears practical and reliable, though further validation in larger populations is warranted.

Our findings align with this target window (Figure 3). The 750 mg group representing the starting dose phase, have a median AUC of 43.5 mg·h/L (IQR: 34.5–53.5). This dose level provides exposure within the target range during the early post-transplant period when the risk of acute rejection is highest. The 500 mg group represents the maintenance phase, where the median AUC was 35 mg·h/L, (IQR: 29–51). However, our data also highlight individual variability and dose–response unpredictability, as illustrated in Figure 2 and Figure 3. Hence, a 750 mg starting dose can yield similar exposure to a 500 mg maintenance dose, which means dose alone does not confirm a specific AUC. Instead, the results likely reflect the intended tapering of steroid and CNI doses over time and the impact of patient-specific clinical factors, such as the factors identified in this study (albumin, BMI, age, and sex). The low R^2^ values observed for the identified covariates indicate that a substantial proportion of variability in MPA exposure remains unexplained, warranting further studies.

While the 30–60 mg·h/L targets are primarily established for the early post-transplant period to prevent acute rejection, there is currently a lack of robust clinical data defining optimal AUC targets for the long-term maintenance phase [35,45,51,52]. 

## 5. Study Limitations

Our study has several limitations. First, MPA-AUC calculations were derived from blood samples collected according to the validated limited sampling strategy (LSS) at three time points: trough, 30 min, and 120 min post-dose. Although MPA-C_max_ is expected near 30 min, 25 samples (24%) exhibited higher concentrations at 120 min, potentially reflecting co-medication effects or enterohepatic recirculation. An additional later blood sampling (e.g., 240 min) could help resolve this discrepancy. Additionally, the absence of measurements for the primary metabolite MPAG limits the depth of pharmacokinetic characterization and precludes analysis of metabolite-mediated variability in MPA exposure. Due to the retrospective nature of the study, we were limited to the data available at the time of sampling. Consequently, variables such as dietary intake and liver function parameters were not consistently recorded and could not be included in the analysis.

As data were collected over an extended period, some patients may have undergone dose adjustments between sampling occasions. Furthermore, repeated measurements within the same patient may introduce intra-individual correlation that is not fully accounted for in the current analysis. A mixed-effects or repeated-measures modeling approach could further address this aspect.

Finally, our analysis did not incorporate liver function parameters, which are known to significantly influence MPA metabolism and could contribute to interindividual variability in drug exposure.

## 6. Conclusions

Implementing LSS-based AUC monitoring—using 30–60 mg·h/L targets for kidney transplant recipients and tailoring exposure to CNI regimen, formulation, and patient-specific factors—could be a pragmatic and feasible approach to optimize and evaluate MMF dosing. Our study found that the patient-specific factors BMI, sex, age, and P-albumin were significant determinants of MPA-AUC, whereas kidney function (eGFR) was not associated with overall exposure. These findings underscore the importance of incorporating individual patient characteristics into MPA pharmacokinetic assessment to support more precise dosing in clinical practice. Larger studies are needed to further clarify the influence of patient-specific factors on MPA-AUC and to evaluate the clinical outcomes of TDM-guided dosing regimens.

## Figures and Tables

**Table 1:** Baseline clinical features and demographics of the patients. Continuous variables (e.g., age, weight, plasma concentrations) are summarized as medians with interquartile ranges [IQR]. Categorical variables (e.g., sex, immunosuppressive regimen) are reported as counts and percentages. Abbreviations: kg, kilograms; BMI, body mass index; eGFR, estimated glomerular filtration rate; PPI, proton pump inhibitor. Biochemical parameters are stratified by first sample (2–3 weeks) and subsequent samples (8–10 weeks). One patient contributed three samples.

Total	All (N = 66)	
Age (years)	52.8 [43–63]	
Sex (male)	46 (69.6%)	
Body weight (kg)	77.5 [69–86]	
BMI (kg/m^2^)	25.5 [23–29]	
Transplantation number		
First transplantation	53 (80.3%)	
Donor		
Deceased	39 (59%)	
Living	37 (41%)	
Co-medication		
Prednisolone	66 (100%)	
Antihypertensive	58 (87.8%)	
PPI	56 (84.8%)	
Tacrolimus	66 (100%)	
Biochemistry	First Sample (N = 66)	Second Sample (N = 36)
B-tacrolimus (µg/L)	10.1 [8.1–12.6]	8.4 [7.6–10.4]
P-creatinine (µmol/L)	139.0 [109.0–163.0]	135.0 [113.0–156.0]
eGFR (mL/min/1.73m^2^)	45.5 [36.3–61.8]	48.0 [39.5–59.5]
P-albumin (g/L)	36.0 [34.0–38.0]	37.5 [35.0–40.0]

**Table 2:** Influence of covariates on the AUC of MPA (N = 103); the influence of each independent variable is elucidated, with *p*-values in bold are regarded as statistically significant.

Predictor/Covariate	Estimate (β) mg·h/L	95% CI mg·h/L	*p*-Value	Adjusted R^2^
Dose (mg/kg)	0.018	0.01–0.03	**<0.001**	—
C_00min_ (mg/L)	8.998	8.13–9.87	**<0.001**	0.830
C_30min_ (mg/L)	1.094	0.55–1.64	**<0.001**	0.230
C_120min_ (mg/L)	4.257	3.52–4.99	**<0.001**	0.600
Donor (dead)	−1.780	−8.11–4.55	0.580	0.090
Sex (male)	−7.750	−14.34–−1.16	**0.022**	0.135
Body weight (kg)	−0.206	−0.43–0.01	0.065	0.115
BMI (kg/m^2^)	−1.094	−2.19–0.00	**0.050**	0.165
Age (years)	−0.282	−0.56–−0.00	**0.049**	0.169
eGFR (mL/min/1.73 m^2^)	0.029	−0.14–0.20	0.736	0.085
P-albumin (g/L)	1.849	1.04–2.66	**<0.001**	0.240
P-creatinine (µmol/L)	−0.025	−0.07–0.03	0.327	0.093
B-tacrolimus (µg/L)	0.187	−0.83–1.20	0.715	0.085
Weeks post-transplant	0.489	−0.27–1.25	0.206	0.102

**Table 3:** Influence of covariates on the trough concentration (pre-dose measurement) of MPA (N = 103); *p*-values in bold are regarded as statistically significant.

Covariate	Estimate (β) mg/L	95% CI mg/L	*p*-Value	Adjusted R^2^
Dose (mg/kg)	0.002	0.000–0.004	**0.040**	0.041
Donor (dead)	−1.780	−8.110–4.550	0.580	0.090
Sex (male)	−0.440	−1.110–−0.230	0.197	0.038
Body weight (kg)	−0.015	−0.037–0.007	0.189	0.056
BMI (kg/m^2^)	−0.137	−0.241–−0.033	**0.011**	0.180
Age (years)	−0.026	−0.054–0.001	0.057	0.140
eGFR (mL/min/1.73 m^2^)	−0.005	−0.022–0.012	0.567	0.042
P-albumin (g/L)	0.177	0.095–0.258	**>0.001**	0.190
P-creatinine (µmol/L)	0.001	−0.004–0.006	0.764	0.040
B-tacrolimus (µg/L)	−0.011	−0.113–0.091	0.829	0.039
Weeks post-transplant	0.0341	−0.042–0.1116	0.378	0.029

**Table 4:** Influence of covariates on the C_30_ (30 min post-dose measurement) of MPA (N = 103); *p*-values in bold are regarded as statistically significant.

Covariate	Estimate (β) mg/L	95% CI mg/L	*p*-Value	Adjusted R^2^
Dose (mg/kg)	0.008	−0.000–0.015	0.052	0.027
Donor (dead)	−2.500	−4.580–−0.420	**0.019**	0.070
Sex (male)	−3.480	−5.660–−1.300	**0.002**	0.107
Body weight (kg)	−0.018	−0.093–0.057	0.630	0.018
BMI (kg/m^2^)	0.111	−0.259–0.482	0.550	0.000
Age (years)	−0.027	−0.122–0.069	0.577	0.005
eGFR (mL/min/1.73 m^2^)	0.036	−0.022–0.092	0.220	0.031
P-albumin (g/L)	0.236	−0.061–0.533	0.118	0.040
P-creatinine (µmol/L)	−0.019	−0.036–−0.003	**0.021**	0.068
B-tacrolimus (µg/L)	0.133	−0.210–0.475	0.444	0.022
Weeks post-dose	−0.007	−0.267–0.252	0.956	0.018

## Data Availability

Data is unavailable due to patient privacy restrictions. Further inquiries can be directed to the corresponding author(s).

## Abbreviations

AUC: Area Under the Curve, TDM: Therapeutic Drug Monitoring, MMF: Mycophenolate Mofetil, MPA: Mycophenolic Acid, AUH: Aarhus University Hospital, LSS: Limited Sampling Strategy, EPJ: Electronic Patient Journal, C: Concentration.
